# Non-linear association between serum levels of vitamins A and B12 and accelerated epigenetic aging

**DOI:** 10.3389/fnut.2025.1599205

**Published:** 2025-07-28

**Authors:** Zhimin Ma, Mingxing An, Weiwei Gong, Xiangyu Chen, Mingbin Liang, Jie Zhang, Xiaofu Du, Feng Lu, Qingfang He, Meng Wang, Jieming Zhong, Ce Sun

**Affiliations:** ^1^Department of Chronic Disease Prevention and Control, Zhejiang Provincial Center for Disease Control and Prevention, Hangzhou, China; ^2^Department of Epidemiology, School of Public Health, Southeast University, Nanjing, China; ^3^Key Laboratory of Systems Health Science of Zhejiang Province, School of Life Science, Hangzhou Institute for Advanced Study, University of Chinese Academy of Sciences, Hangzhou, China

**Keywords:** accelerated epigenetic aging, biological aging, vitamin A, vitamin B12, non-linear association

## Abstract

**Background:**

Serum vitamins A and B12, as essential micronutrients, play pivotal roles in maintaining physiological homeostasis; however, the association between these vitamins and aging remains unclear. Therefore, this study aims to investigate potential threshold effects of these nutrients on accelerated biological aging using multidimensional DNA methylation biomarkers.

**Methods:**

This study included 2,530 participants with DNA methylation data from the National Health and Nutrition Examination Survey 1999–2000 and 2001–2002. Two age acceleration metrics, derived from epigenetic clocks (PhenoAge and GrimAge), were calculated as the residuals obtained by regressing the epigenetic clock estimates on chronological age. Multivariable logistic regression models were used to analyze the association of vitamins A and B12 with epigenetic clocks. Additionally, generalized additive models and two-piecewise logistic regression were used to explore the non-linear relationships between vitamins A and B12 and epigenetic clocks.

**Results:**

Compared to the first quintile of vitamin A, the odds ratios (ORs) for PhenoAge acceleration in the next four quintiles were 1.24 (0.93–1.65), 1.04 (0.78–1.37), 0.95 (0.71–1.27), and 1.51 (1.13–2.01), respectively. No linear associations were found between vitamin B12 and PhenoAge acceleration, nor between vitamins A and B12 and GrimAge acceleration. However, the generalized additive model showed significant non-linear associations between serum levels of vitamins A and B12 and PhenoAge acceleration, with inflection points at 71.5 and 488.0 pg/mL, respectively. In addition, a non-linear association was observed between serum levels of vitamin A with GrimAge acceleration, with an inflection point at 71.8 μg/dL. Two-piecewise logistic regression also indicated that higher vitamin B12 delayed aging, while higher vitamin A accelerated aging. Sensitivity analyses showed a similar non-linear association between vitamins A and B12 and HannumAge acceleration.

**Conclusion:**

This study suggests that higher vitamin A concentrations may be related to an increased risk of aging, while adequate vitamin B12 intake may offer protective benefits against epigenetic changes associated with aging.

## 1 Introduction

According to the report of the World Health Organization (WHO), the population aged 60 years and above currently exceeds 1 billion. By 2050, this number is expected to double, reaching 2.1 billion globally (1). As the challenges associated with an aging population intensify, the importance of healthy aging has become increasingly critical. Aging is a natural physiological process in most living organisms, characterized by a time-dependent decline in body function. Traditionally, chronological age has been used as the primary measure of aging. However, recent studies showed that biological age could more accurately reflect a person’s aging status compared to chronological age, as the latter did not consider an individual’s health status (2–4). Biological aging could raise the accumulation of molecular changes or “hallmarks” that deteriorate the function and resilience of tissues and organs, eventually leading to death and age-related diseases such as cardiovascular disease and cancer (3, 4).

Recent evidence from human and mouse studies demonstrates that DNA methylation-based (DNAm) biomarkers satisfy the formerly elusive criteria of a molecular biomarker of aging and thus serve as vital inputs for constructing epigenetic clocks (5). These clocks can accurately predict biological aging by accounting for both genetic predispositions and environmental factors (6). HannumAge and HorvathAge were the “first-generation” clocks, which are primarily based on chronological age. In contrast, “second-generation” clocks, such as GrimAge and PhenoAge, emphasize health and mortality outcomes (7). Among these, PhenoAge and GrimAge have stronger evidence of association with morbidity and mortality and with risk factors for shorter and less healthy lives (8). When the epigenetic clock exceeds chronological age, individuals are considered to be in the state of accelerated aging, which is linked to an increased risk of diseases (6, 9). However, few studies have explored the factors that affect accelerated epigenetic aging, especially the association of nutrients with aging (7).

Vitamins are essential nutrients that are significantly associated with aging-related diseases. Vitamin A, a crucial fat-soluble vitamin, is an indispensable dietary micronutrient essential for human health and development, supporting physiological processes, including immune function, cellular growth, and metabolic regulation (10, 11). Previous studies have shown that adults in high-income countries often use dietary supplements containing vitamin A, which increases the risk of intake above tolerable upper intake levels (12). However, vitamin A deficiency has been linked to obesity and carcinogenesis in the oral cavity (13, 14). In addition, vitamin B12, a water-soluble vitamin, plays a critical role in physiological functions such as DNA synthesis and methylation, inflammatory response, and neurological function. Previous studies have shown that vitamin B12 deficiency could elevate homocysteine levels, thereby increasing the risk of cardiovascular disease (15). Moreover, vitamin B12 deficiency was associated with neurological sequelae such as peripheral neuropathy, subacute combined degeneration of the spinal cord, cognitive decline, and psychiatric disturbances (16, 17). However, few studies explored the association between vitamins and epigenetic aging.

Previous studies have shown that serum levels of vitamin D are negatively associated with aging (18–22). Limited evidence exists regarding the associations between serum levels of vitamins A and B12 and aging. While most prior investigations examined linear associations of vitamins with health outcomes (e.g., mortality or disease risk), the optimal concentration ranges for these vitamins remain unknown. For example, previous studies showed that serum levels of vitamin A <30 μg/dL or >80 μg/dL may indicate a high risk of subsequent mortality (23). Additionally, both low (<369.1 pg/mL) and high (≥506.1 pg/mL) serum vitamin B12 levels were associated with a higher risk of cardiovascular disease (CVD) mortality in diabetes patients (24). To fill these knowledge gaps, we examined the non-linear associations between serum vitamins A and B12 concentrations and GrimAge and PhenoAge acceleration, based on the National Health and Nutrition Examination Survey (NHANES). Furthermore, the inflection points of these associations were also determined.

## 2 Materials and methods

### 2.1 Study design and sample

Participants of this cross-sectional study came from the NHANES, a nationally representative sample of the US population, with methodological details reported previously (25). Briefly, the NHANES was conducted by the National Center for Health Statistics of the Centers for Disease Control and Prevention. All participants were recruited from the civilian, non-institutionalized household population of the United States between 1988 and 2018. Baseline information was collected using standardized questionnaires and home interviews, and blood samples were collected and tested following standard procedures.

The data for our study were obtained from the two-cycle data of NHANES (1999–2000 and 2001–2002). After excluding the participants with missing DNAm PhenoAge (*N* = 18,472) and serum levels of vitamin A (*N* = 5) and vitamin B12 (*N* = 2) at baseline, 2,527 and 2,530 participants were used to calculate the association of serum levels of vitamins A and vitamin B12 with epigenetic clocks, respectively (Supplementary Figure 1).

### 2.2 Serum levels of vitamins A and B12 assessment

The serum levels of vitamins A and B12 were measured using high-performance liquid chromatography with photodiode array detection in the NHANES 1999–2000 and 2001–2002. The NHANES quality assurance and quality control protocols complied with the 1988 Clinical Laboratory Improvement Act Mandates.

### 2.3 Accelerated epigenetic aging assessment

This study evaluated accelerated epigenetic aging through DNAm-based epigenetic clocks. DNA methylation profiling was performed on purified whole blood samples obtained from participants in NHANES during the 1999–2000 and 2001–2002 cycles. Genome-wide DNAm levels were quantified using the Illumina Infinium MethylationEPIC BeadChip v1.0 (Illumina, San Diego, CA, United States). Data preprocessing, normalization, and quality control procedures followed established protocols, as detailed in NHANES (26). Two widely validated epigenetic clocks, including PhenoAge (27) and GrimAge (28) were computed to capture distinct dimensions of biological aging. PhenoAge and GrimAge incorporate clinical biomarkers and mortality-related predictors to quantify morbidity- and mortality-associated aging trajectories (29). Age acceleration, a measure of epigenetic aging discordance, was derived for each clock by regressing chronological age against its corresponding epigenetic clock estimate. Accelerated epigenetic aging was defined as the positive age acceleration, indicating that the epigenetic clock was older than chronological age.

### 2.4 Covariate assessment

As both vitamins (A and B12) and accelerated epigenetic aging could be influenced by sex, age, ethnicity, socioeconomic factors [education and poverty income ratio (PIR)], lifestyle factors [smoking and alcohol use], body mass index (BMI), and history of disease [diabetes, cancer, coronary heart disease (CHD) and stroke]. The history of disease was defined based on self-reported diagnoses by doctors or the use of drugs associated with those diseases.

### 2.5 Statistical analyses

To reduce the effect of false positives, our study used winsorization to adjust for outliers prior to log-transformation (30). The outliers were capped by the 5th percentile (Q5) or 95th percentile (Q95) of vitamin levels. The chi-squared test was used to compare baseline categorical variables by quintiles of serum levels of vitamins A and B12, and analysis of variance (ANOVA) or Kruskal–Wallis rank sum test for continuous variables. Logistic regression was used to calculate odds ratios (ORs) and 95% confidence intervals (CIs) for the association of serum levels of vitamins A and B12 with accelerated epigenetic aging. Model 1 was the crude model without adjustment. In multivariable analyses, model 2 was adjusted for sex, age, ethnicity, and PIR, and model 3 additionally adjusted for lifestyle factors (smoking and alcohol use), BMI, and comorbidities (history of diabetes, cancer, stroke, and CHD).

Furthermore, potential non-linear associations between serum levels of vitamins A and B12 and accelerated epigenetic aging were explored using logistic regression with generalized additive model and smoothed curve fitting (penalized spline method). Vitamins A and B12 were included in the models as continuous, log2-transformed, and scaled values. A recursive algorithm was then employed to estimate the inflection points from these non-linear associations, and a two-segment logistic regression model based on inflection points was used.

### 2.6 Sensitivity analysis

To assess potential effect modification, a log-likelihood ratio test was used to calculate the model fit by comparing models with and without interaction terms of vitamins A and B12 with selected potential effect modifiers. When significant interactions were found, we conducted subgroup analyses by the possible effect modifiers. To assess the robustness of our findings, we used HorvathAge and HannumAge acceleration, which have been extensively validated in previous studies. In addition, we analyzed the association between serum vitamin quantiles and epigenetic age acceleration, with model fit assessed using Akaike Information Criterion (AIC) values. Two DNAm ages were trained on chronological age and reflect mitotic and tissue-specific aging processes (31, 32). Statistical analysis was performed using R (version 4.4.1; R Core Team, Vienna, Austria). Two-sided *p* < 0.05 were considered statistically significant.

## 3 Results

### 3.1 Participant characteristics

Table 1 shows that at baseline compared with those in the lowest quintile (Q1) of vitamin A level, participants in the highest quintile (Q5) were more likely to be men, older and non-Hispanic White, had higher family income, more smoking and alcohol use, were less likely to be obese (all *p* < 0.05). They had a higher prevalence of diabetes, cancer, and CVD (all *p* < 0.05), but similar prevalence of stroke (*p* = 0.28). In contrast, participants in the highest quintile (Q5) of vitamin B12 were more likely to be women and non-Hispanic Black, with lower smoking and alcohol use. At the same time, age, income, and prevalence of cancer and CHD were similar.

**Table 1:** Baseline characteristics of participants according to serum levels of vitamins A and vitamin B12^a^.

Characteristic	Serum vitamins levels					*p* value
	Q1	Q2	Q3	Q4	Q5	
Vitamin A						
Sex, women	305 (60.2)	243 (48.2)	257 (50.8)	235 (46.6)	205 (40.5)	<0.01
Age, mean (SD), years	65.6 (9.9)	65.2 (10.1)	65.7 (10.3)	66.9 (10.2)	67.4 (9.8)	<0.01
Race/ethnicity						<0.01
Mexican American	208 (41.0)	182 (36.1)	122 (24.1)	117 (23.2)	90 (17.8)	
Other Hispanic	42 (8.3)	44 (8.7)	32 (6.3)	22 (4.4)	23 (4.5)	
Non-Hispanic White	96 (18.9)	155 (30.8)	228 (45.1)	266 (52.8)	280 (55.3)	
Non-Hispanic Black	146 (28.8)	111 (22.0)	100 (19.8)	84 (16.6)	96 (19.0)	
Other race-including multi-racial	15 (3.0)	12 (2.4)	24 (0.9)	15 (3.0)	17 (3.4)	
Family PIR, mean (SD)	2.1 (1.5)	2.4 (1.5)	2.7 (1.6)	2.8 (1.6)	2.8 (1.7)	<0.01
Smoking status						0.02
≥100 cigarettes in life	255 (50.3)	260 (51.6)	264 (52.3)	275 (54.7)	301 (59.5)	
<100 cigarettes in life	250 (49.3)	242 (48.0)	241 (47.7)	228 (45.3)	205 (40.5)	
Not recorded	2 (0.4)	2 (0.4)	0 (0.0)	0 (0.0)	0 (0.0)	
Alcohol use						<0.01
≥12 alcohol drinks/year	255 (53.6)	293 (62.6)	313 (64.0)	309 (64.2)	333 (68.1)	
<12 alcohol drinks/year	221 (46.4)	175 (39.4)	176 (36.0)	172 (35.8)	156 (31.9)	
BMI, mean (SD), kg/m^2^	29.8 (6.4)	28.7 (5.8)	28.7 (6.0)	27.9 (5.3)	28.2 (5.4)	<0.01
History of diabetes	112 (22.1)	82 (16.3)	95 (18.8)	100 (19.8)	123 (24.3)	0.02
History of cancer	52 (10.3)	61 (12.1)	80 (15.8)	62 (12.3)	90 (17.8)	<0.01
History of stroke	25 (4.9)	22 (4.4)	31 (6.1)	34 (6.77)	36 (7.1)	0.28
History of CHD	41 (8.1)	33 (6.5)	46 (9.1)	45 (8.9)	77 (15.2)	<0.01
Vitamin B12						
Sex, women	218 (43.1)	219 (43.4)	234 (46.2)	257 (50.7)	317 (63.3)	<0.01
Age, mean (SD), years	67.1 (10.3)	66.1 (10.0)	65.3 (10.0)	65.9 (9.8)	66.3 (10.1)	0.07
Race/ethnicity						<0.01
Mexican American	140 (27.7)	136 (26.9)	160 (31.6)	131 (25.8)	152 (30.3)	
Other Hispanic	35 (6.9)	38 (7.5)	40 (7.9)	25 (4.9)	25 (5.0)	
Non-Hispanic White	215 (42.5)	239 (47.3)	203 (40.1)	194 (38.3)	172 (34.3)	
Non-Hispanic Black	98 (19.4)	79 (15.7)	87 (17.2)	137 (27.0)	136 (27.1)	
Other race-including multi-racial	18 (3.5)	13 (2.6)	16 (3.2)	20 (4.0)	16 (3.3)	
Family PIR, mean (SD)	2.6 (1.6)	2.6 (1.6)	2.6 (1.6)	2.6 (1.6)	2.6 (1.6)	0.99
Smoking status						<0.01
≥100 cigarettes in life	280 (55.6)	295 (58.4)	300 (59.3)	244 (48.1)	235 (46.9)	
<100 cigarettes in life	222 (44.0)	210 (41.6)	205 (40.5)	263 (51.9)	265 (52.9)	
Not recorded	2 (0.4)	0 (0.0)	1 (0.2)	0 (0.0)	1 (0.2)	
Alcohol use						<0.01
≥12 alcohol drinks/year	322 (67.1)	317 (66.0)	308 (63.8)	300 (62.1)	254 (53.5)	
<12 alcohol drinks/year	158 (32.9)	163 (34.0)	175 (36.2)	183 (37.9)	221 (46.5)	
BMI, mean (SD), kg/m^2^	29.4 (6.4)	29.0 (5.6)	28.8 (5.7)	28.6 (5.8)	27.7 (5.6)	<0.01
History of diabetes	86 (17.0)	77 (15.2)	111 (21.9)	117 (23.1)	121 (24.2)	<0.01
History of cancer	76 (15.0)	69 (13.7)	69 (13.7)	64 (12.6)	66 (13.2)	0.85
History of stroke	23 (4.5)	35 (6.9)	30 (5.9)	34 (6.7)	26 (5.2)	0.45
History of CHD	49 (9.7)	47 (9.3)	48 (9.5)	51 (10.1)	47 (9.4)	0.99

BMI, body mass index; CHD, coronary heart disease; PIR, poverty income ratio; SD, standard deviation.

a Data are presented as number (percentage) of study participants unless otherwise indicated.

### 3.2 Association of vitamins A and B12 with accelerated epigenetic aging

Table 2 shows that compared to the first quintile of vitamin A, the fully adjusted OR (95% CI) of PhenoAge acceleration for the second to fifth quintiles were 1.24 (95% CI: 0.93–1.65), 1.04 (95% CI: 0.78–1.37), 0.95 (95% CI: 0.71–1.27), and 1.51 (95% CI: 1.13–2.01), respectively. Although we found no significant linear association of serum levels of vitamin B12 with PhenoAge acceleration [Q1: reference; Q2: OR = 1.20, 95% CI: 0.91–1.58; Q3: OR = 1.36, 95% CI: 1.03–1.80; Q4: OR = 0.98, 95% CI: 0.74–1.29; and Q5: OR = 0.88, 95% CI: 0.66–1.16; *p* for trend = 0.16], the trend of the OR values suggested a potential non-linear dose–response association. Similarly, we found no significant linear association of serum levels of vitamins A and B12 with GrimAge acceleration (*p* for trend = 0.23 and 0.26, respectively). However, nonmonotonic trends in ORs across quintiles suggested possible threshold or biphasic effects.

**Table 2:** Association between serum levels of vitamin and epigenetic accelerated aging.

Variable	Serum vitamins levels					*p* for trend
	Q1	Q2	Q3	Q4	Q5	
PhenoAge acceleration						
Vitamin A						
Crude OR (95% CI)	1.00	1.13 (0.88–1.13)	0.99 (0.77–1.45)	0.91 (0.71–1.16)	1.36 (1.06–1.74)^*^	0.17
Adjusted OR (95% CI)^a^	1.00	1.25 (0.95–1.63)	1.01 (0.77–1.32)	0.99 (0.75–1.30)	1.56 (1.18–2.05)^**^	0.03
Adjusted OR (95% CI)^b^	1.00	1.24 (0.93–1.65)	1.04 (0.78–1.37)	0.95 (0.71–1.27)	1.51 (1.13–2.01)^**^	0.04
Vitamin B12						
Crude OR (95% CI)	1.00	1.25 (0.98–1.60)	1.38 (0.98–1.60)	1.01 (0.79–1.29)	0.91 (0.71–1.17)	0.17
Adjusted OR (95% CI)^a^	1.00	1.17 (0.90–1.53)	1.38 (1.06–1.80) ^*^	0.98 (0.75–1.27)	0.87 (0.67–1.14)	0.15
Adjusted OR (95% CI)^b^	1.00	1.20 (0.91–1.58)	1.36 (1.03–1.80) ^*^	0.98 (0.74–1.29)	0.88 (0.66–1.16)	0.16
GrimAge Acceleration						
Vitamin A						
Crude OR (95% CI)	1.00	0.93 (0.73–1.20)	0.93 (0.73–1.20)	0.81 (0.63–1.04)	1.19 (0.93–1.52)	0.46
Adjusted OR (95% CI)^a^	1.00	0.81 (0.61–1.08)	0.87 (0.65–1.15)	0.70 (0.52–0.94) ^*^	1.00 (0.75–1.34)	0.73
Adjusted OR (95% CI)^b^	1.00	0.78 (0.56–1.10)	0.83 (0.60–1.16)	0.61 (0.43–0.86) ^*^	0.87 (0.62–1.22)	0.23
Vitamin B12						
Crude OR (95% CI)	1.00	0.80 (0.63–1.03)	0.93 (0.72–1.19)	0.67 (0.52–0.86) ^**^	0.66 (0.51–0.85) ^**^	<0.01
Adjusted OR (95% CI)^a^		0.81 (0.61–1.07)	0.99 (0.75–1.32)	0.71 (0.54–0.94) ^*^	0.80 (0.60–1.07)	0.08
Adjusted OR (95% CI)^b^	1.00	0.82 (0.59–1.13)	0.93 (0.67–1.29)	0.75 (0.54–1.04)	0.84 (0.60–1.18)	0.26

a Adjusted for sex, PIR, and ethnicity.

b Additionally adjusted for smoking status, alcohol use, BMI, and history of diabetes, cancer, stroke, and CHD.

OR, odds ratio; CI, confidence interval; BMI, body mass index; CHD, coronary heart disease; PIR, poverty income ratio. * *p* < 0.05; ** *p* < 0.01; *** *p* < 0.001.

### 3.3 The detection of non-linear association

**Figure 1:**
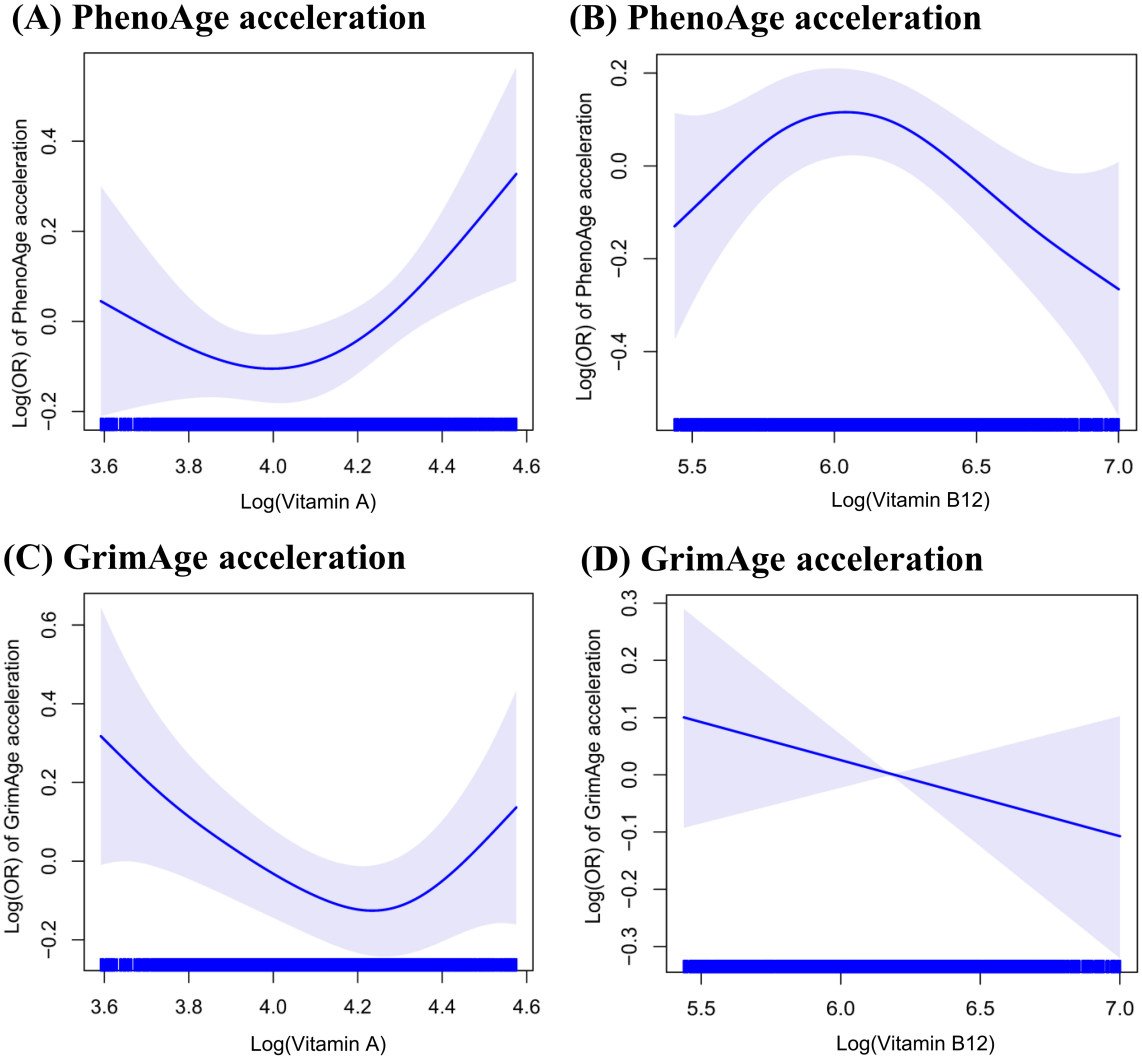
The non-linear association of serum levels of vitamins A and B12 concentrations with accelerated epigenetic aging.

Figure 1 shows that the generalized additive model revealed a non-linear association of serum levels of vitamin A (*p* for non-linear = 0.02 for PhenoAge acceleration and *p* for non-linear = 0.01 for GrimAge acceleration) and serum levels of vitamin B12 (*p* for non-linear = 0.03 for PhenoAge acceleration) with accelerated epigenetic aging. The inflection points of serum levels of vitamins A and B12 with PhenoAge acceleration were 71.5 μg/dL and 488.0 pg/mL, respectively. In addition, the points at which the curve crossed the horizontal vector were similar for the PhenoAge acceleration and GrimAge acceleration (71.8 μg/dL for vitamin A). However, no significant non-linear association was found between vitamin B12 and GrimAge acceleration (*p* for non-linear = 0.30).

Furthermore, two-piecewise logistic regression shows similar results (all *p* for log-likelihood ratio < 0.05, Table 3). Meanwhile, if the serum vitamin A concentration is ≥71.5 μg/dL, each standard deviation (SD) raises the risk of PhenoAge acceleration by 26% (OR = 1.26, 95% CI: 1.06–1.51). However, per 1 SD increase in serum levels of vitamin B12 was associated with a 14% lower risk of PhenoAge acceleration when the concentration was ≥488.0 pg/mL (OR = 0.86, 95% CI: 0.75–0.98). When serum levels of vitamins A and B12 were <71.5 μg/dL and 488.0 pg/mL, respectively, no associations were observed between those vitamins and PhenoAge acceleration (OR = 0.96, 95% CI: 0.86–1.07 and OR = 1.07, 95% CI: 0.95–1.22, respectively). If the serum vitamin A concentration is ≥71.8 μg/dL, each SD raises the risk of GrimAge acceleration by 33% (OR = 1.33, 95% CI: 1.09–1.62). However, no associations were observed between vitamin A and GrimAge acceleration (OR = 0.89, 95% CI: 0.78–1.01).

**Table 3:** Threshold effect analysis of serum levels of vitamin on epigenetic accelerated aging.

Variable	Adjusted OR (95% CI)	*p*
PhenoAge Acceleration		
Vitamin A		
Fitting by the logistic model	1.10 (1.01–1.21)	0.03
Fitting by the two-piecewise logistic model		
Threshold value		
≥71.5 ug/dL	1.26 (1.06–1.51)	<0.01
<71.5 ug/dL	0.96 (0.86–1.07)	0.42
*p* for log-likelihood ratio		0.02
Vitamin B12		
Fitting by the logistic model	0.93 (0.85–1.02)	0.10
Fitting by the two-piecewise logistic model		
Threshold value		
≥488.0 pg/mL	0.86 (0.75–0.98)	0.02
<488.0 pg/mL	1.07 (0.95–1.22)	0.26
*p* for log-likelihood ratio		0.02
GrimAge Acceleration		
Vitamin A		
Fitting by the logistic model	0.96 (0.86–1.07)	0.49
Fitting by the two-piecewise logistic model		
Threshold value		
≥71.8 ug/dL	1.33 (1.09–1.62)	<0.01
<71.8 ug/dL	0.89 (0.78–1.01)	0.08
*p* for log-likelihood ratio		<0.01
Vitamin B12		
Fitting by the logistic model	0.96 (0.86–1.07)	0.45
Fitting by the two-piecewise logistic model		
Threshold value		
\	\	\
\	\	\
*p* for log-likelihood ratio		\

OR, odds ratio; CI, confidence interval; PIR, poverty income ratio; BMI, body mass index; CHD, coronary heart disease.

The model was adjusted for sex, ethnicity, PIR, smoking status, alcohol use, BMI, and history of diabetes, cancer, stroke, and CHD.

### 3.4 Subgroup analysis and sensitivity analysis

**Figure 2:**
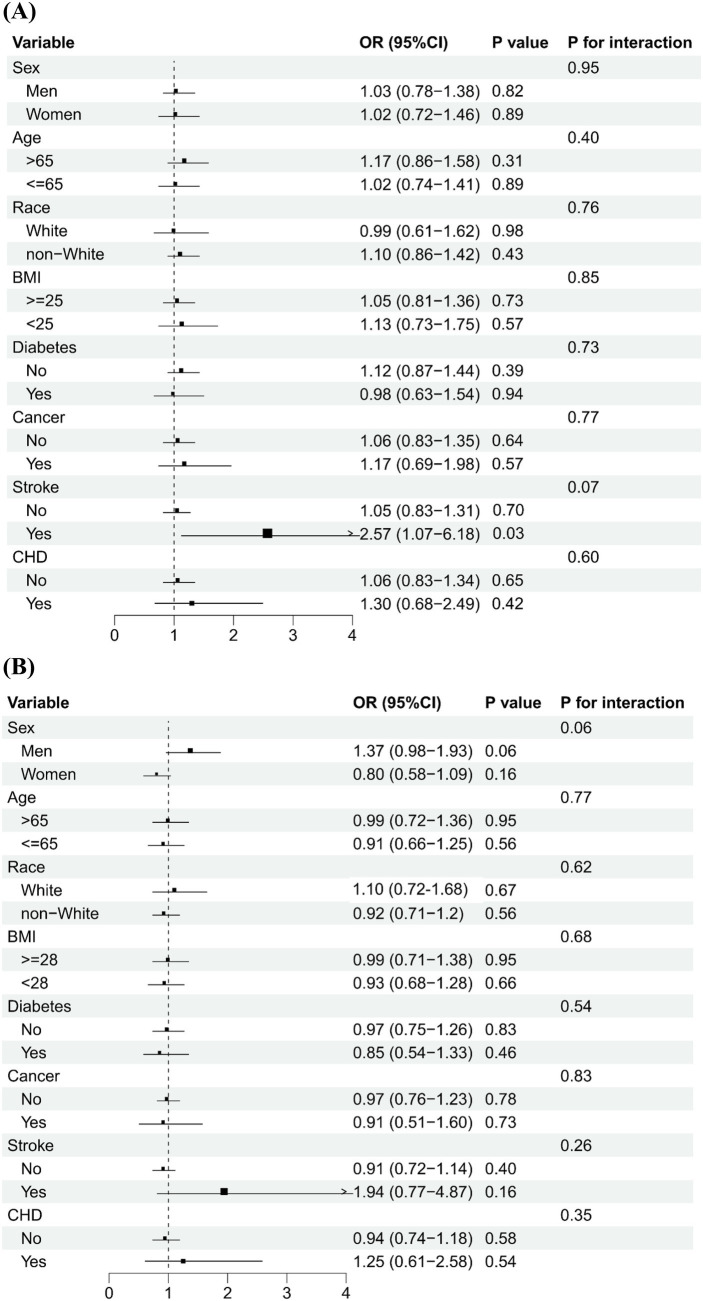
Subgroup analysis of the association of higher levels of serum vitamins A and B12 with PhenoAge acceleration.

**Figure 3:**
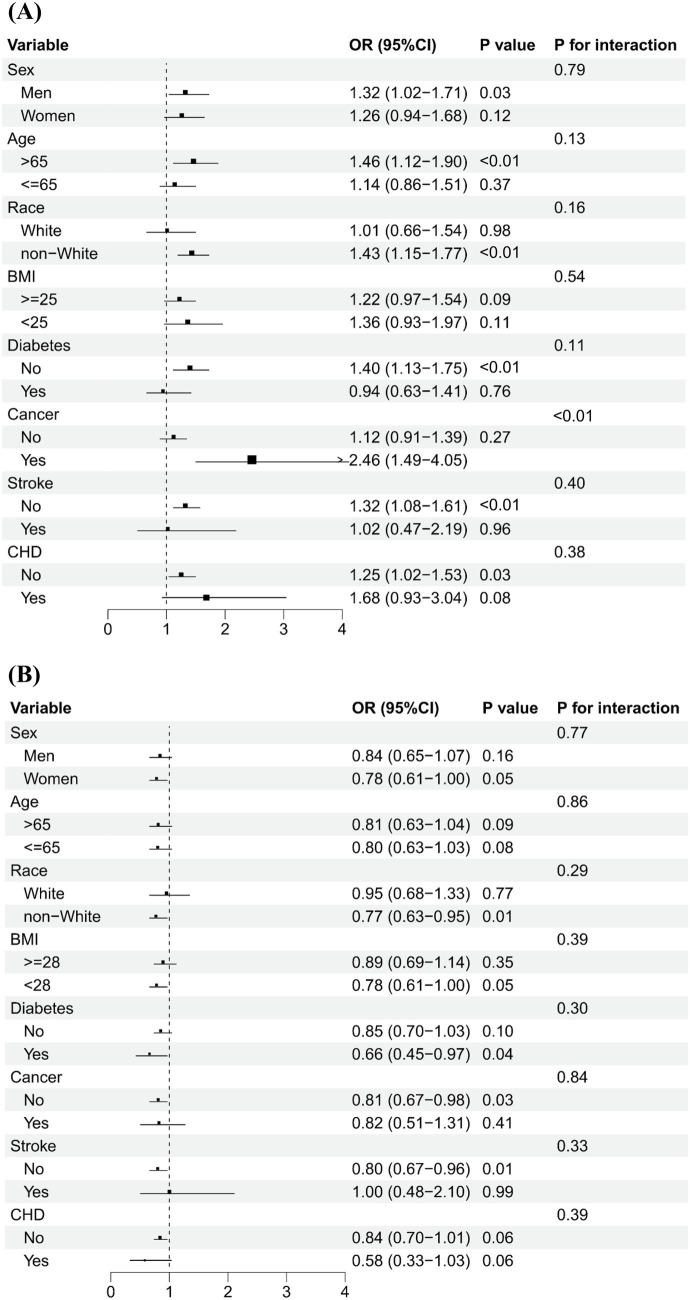
Subgroup analysis of the association of high levels of serum vitamins A and B12 with GrimAge acceleration.

Figure 2 shows that serum levels of vitamins A and B12 were categorized into low and high groups using a cut-off value of 71.5 μg/dL and 488.0 pg/mL, respectively. The association of high vs. low serum vitamins A and B12 concentrations on PhenoAge acceleration was consistent across various subgroups by age, sex, ethnicity, BMI, and history of diabetes, stroke, and CHD. No significant interactions were observed between serum levels of vitamins (A and B12) and subgroup variables related to epigenetic acceleration of aging (PhenoAge acceleration and GrimAge acceleration), except in cancer subgroup analyses. The positive association between serum levels of vitamin A and PhenoAge Acceleration was stronger in participants with cancer than in participants without cancer (*p* for interaction <0.01), with the OR being 2.46 (1.49–4.05) and 1.12 (0.91–1.39), respectively. Figure 3 shows that the direction of the association between vitamins A and B12 and GrimAge was almost consistent with the aforementioned association, although it was not statistically significant.

In addition, the associations between serum levels of vitamins A and B12 and HorvathAge acceleration or HannumAge acceleration show similar results. Supplementary Table 1 shows that compared to the first quintile of vitamin A, the highest quintile was associated with higher risks of HorvathAge acceleration (OR = 1.44, 95% CI: 1.07–1.92) and HannumAge acceleration (OR = 1.36, 95% CI: 1.01–1.83). This study also showed no associations between vitamin B12 and HorvathAge acceleration or HannumAge acceleration. Supplementary Table 2 shows consistent associations between serum vitamin quartiles and epigenetic age acceleration in the quartile-based model. Compared to the quartile-based model, the quintile-based model yielded higher AIC values for the association between vitamin A and epigenetic age acceleration (PhenoAge Acceleration: 2,826.80 vs. 2,829.43; GrimAge Acceleration: 2,368.77 vs. 2,369.52), as well as for vitamin B12 (PhenoAge Acceleration: 2,835.98 vs. 2,831.86; GrimAge Acceleration: 2,366.98 vs. 2,366.00). Supplementary Figure 2 shows similar non-linear associations of vitamins A and B12 with HannumAge acceleration (*p* for non-linear = 0.04 and 0.02). Although no significant non-linear associations of those vitamins with HorvathAge acceleration (*p* for non-linear = 0.09 and 0.21 for vitamins A and B12, respectively), high vitamin A and low vitamin B12 concentrations may be associated with HorvathAge acceleration according to the plot of the generalized additive model.

## 4 Discussion

This study found significant non-linear associations between serum levels of vitamins A and B12 and accelerated epigenetic aging. Specifically, higher serum vitamin B12 concentrations (≥488.0 pg/mL) were associated with delayed PhenoAge acceleration. Conversely, higher serum vitamin A concentrations (≥71.5 μg/dL) were linked to accelerated PhenoAge, GrimAge, and HannumAge. Compared with the results of logistic regression between vitamins and biological aging, these results could inform recommendations for optimal serum vitamin concentrations to delay biological aging and have substantial public health implications.

### 4.1 Comparison with previous studies

Previous studies have primarily focused on the association of serum vitamins and mortality risk, as well as aging-related diseases. For example, Chen et al. (33) demonstrated that serum vitamin D deficiency was associated with an increased risk of dementia, while Xiao et al. (34) revealed an L-shaped relationship between vitamin D levels and mortality. However, few studies explored the association between serum vitamins and aging. We searched the PubMed database using the keywords “serum vitamin” and “aging” or “biological age” up to 29 May 2025 and found seven studies exploring the association between vitamins and aging (18–22, 35, 36). Nevertheless, only one study has reported the association of serum B12 levels with phenotypic aging measured by chemistry biomarkers. Although similar results were found, this study did not further investigate the non-linear association of serum B12 with phenotypic aging (35). Additionally, some studies have found an association between serum levels of vitamins A and B12 and age-related diseases, which partially supports our findings (37, 38). For example, one cohort study showed that serum levels of vitamin A were also positively associated with the development of CVD (38). Conversely, low serum vitamin B12 concentrations were associated with higher risks of age-related diseases, such as Alzheimer’s disease and metabolic syndrome (39, 40). Accelerated epigenetic aging provides a more comprehensive assessment of an individual’s overall health status than age-related diseases alone (41). This holistic evaluation allows for timely interventions and the potential to delay the onset of diseases (42).

Furthermore, the clinical reference ranges for vitamins A and B12 are typically above 24.08 μg/dL and 270.8 pg/mL, respectively. These references are disease-defined thresholds. However, optimal biological ranges and the prevention level for aging and age-related disease remain undefined. Therefore, this study further identified a non-linear association of serum levels of vitamin A with PhenoAge, GrimAge, and HannumAge acceleration, and of serum levels of vitamin B12 with PhenoAge acceleration. These results are partially consistent with previous studies (23, 43, 44). For instance, Min et al. (23) observed that serum levels of vitamin A < 30 μg/dL or A > 80 μg/dL indicate a higher risk of subsequent mortality. Liu et al. (24) reported that both low (<369.1 pg/mL) and high (≥506.1 pg/mL) serum levels of vitamin B12 were associated with increased CVD mortality risk in diabetes patients. A cross-sectional study showed that the relationship between circulating vitamin B12 and *α*-Klotho in American adults was inverted U-shaped, which is consistent with the present results (36). While previous studies indicate that serum levels of vitamins A and B12 may reduce premature mortality, they failed to identify protective concentration ranges for health outcomes. In contrast, the present study suggested that the potential optimal concentrations of serum levels of vitamins A and B12 to delay accelerated epigenetic aging were <71.5 μg/dL and ≥488.0 pg/mL, respectively.

### 4.2 Possible explanations

A previous study has shown that vitamin A induces epigenetic changes in monocytes (45). Moreover, excess serum levels of vitamin A could also increase the transport of retinol-binding protein 4 (RBP4), subsequently increasing the risk of CHD, stroke, metabolic syndrome, and cardiovascular risk factors, including triglycerides and hypertension (46–51). Conversely, previous studies found that vitamin B12 deficiency is positively associated with inflammatory factors [such as C-reactive protein (CRP) and interleukin 6 (IL-6)] (52, 53), and leads to metabolic syndrome onset and an increase in cardiovascular risk factors (40, 54). Furthermore, the α-Klotho (known for inhibiting cellular senescence) is a hallmark of aging (3), and it can decelerate the aging process in both animal and human studies (55). One study reported a positive relationship between serum levels of vitamin B12 (<1,020 pg/mL) and α-Klotho concentration (36). Thus, maintaining appropriate serum vitamins A and B12 concentrations may help mitigate accelerated epigenetic aging.

## 5 Study limitations

The present study had some limitations. First, the results of the association analysis between vitamins and the methylation clock are not entirely consistent, and there is no gold standard for measuring biological aging. Recent studies have demonstrated that PhenoAge can effectively predict age-related diseases and mortality in large populations (56). Second, despite adjusting for 11 potential confounders, the lack of adjustment for dietary and total energy intake may have attenuated the observed association toward the null. Third, the sample size may have been insufficient to detect subtle associations, and recall bias could have led to underestimation of the association. Fourth, because of the cross-sectional design, causal relationships cannot be established based on our findings. Further prospective studies are warranted to confirm and clarify this relationship. Fifth, due to a lack of genetic and multiomics data, further studies are warranted to explore the potential mechanism of vitamins A and B12 with epigenetic aging. Finally, although no significant interaction was observed between the vitamins and ethnicity on accelerated epigenetic aging, the generalizability of our findings to ethnic populations should be approached with caution due to potential representativeness limitations.

## 6 Conclusion

This study identified biphasic effects of serum levels of vitamins A and B12 on accelerated epigenetic aging. The findings suggest that higher vitamin A levels may be associated with an increased aging risk, whereas adequate vitamin B12 may offer protective benefits against epigenetic changes associated with aging. This study offers promising insights for developing preventive public health strategies and dietary recommendations to delay accelerated epigenetic aging; however, validation in large-scale clinical trials is necessary.

## Data Availability

The datasets presented in this article are not readily available because this study analyzed datasets that are publicly accessible. Requests to access the datasets should be directed to the NHANES repository. This data can be found at: https://www.cdc.gov/nchs/nhanes/.
