# Supplementary material for: Non-linear association between serum levels of vitamins A and B12 and accelerated epigenetic aging
Source: Front Nutr. 2025 Jul 28;12:1599205. doi: 10.3389/fnut.2025.1599205 (PMC12336032; doi:10.3389/fnut.2025.1599205)
Supplement: Supplementary file 1 [file Table_1.docx]

**Supplementary Table 1** Association of serum vitamins with HorthAge acceleration and HannumAge acceleration

|  | **Serum vitamins levels** | | | | | ***P* for trend** |
| --- | --- | --- | --- | --- | --- | --- |
|  | **Q1** | **Q2** | **Q3** | **Q4** | **Q5** |  |
| **HorvathAge acceleration** |  |  |  |  |  |  |
| Vitamin A |  |  |  |  |  |  |
| Crude OR (95%CI) | 1.00 | 1.47 (1.15 - 1.89) ^**^ | 1.35 (1.06 - 1.74) ^*^ | 1.32 (1.03 - 1.69) ^*^ | 1.49 (1.17 - 1.92) ^**^ | 0.01 |
| Adjusted OR (95%CI) ^a^ | 1.00 | 1.51 (1.15 - 1.98) ^**^ | 1.28 (0.98 - 1.68) | 1.32 (1.01 - 1.74) ^*^ | 1.41 (1.07 - 1.85) ^*^ | 0.09 |
| Adjusted OR (95%CI) ^b^ | 1.00 | 1.50 (1.12 - 2.00) ^**^ | 1.28 (0.96 - 1.69) | 1.28 (0.95 - 1.71) | 1.44 (1.07 - 1.92) ^*^ | 0.10 |
| Vitamin B12 |  |  |  |  |  |  |
| Crude OR (95%CI) | 1.00 | 1.22 (0.96 - 1.56) | 1.19 (0.93 - 1.53) | 0.94 (0.74 o 1.21) | 0.93 (0.73 - 1.20) | 0.17 |
| Adjusted OR (95%CI) ^a^ | 1.00 | 1.15 (0.88 - 1.50) | 1.29 (0.99 - 1.69) | 0.95 (0.73 - 1.24) | 1.01 (0.77 - 1.32) | 0.59 |
| Adjusted OR (95%CI) ^b^ | 1.00 | 1.17 (0.89 - 1.56) | 1.26 (0.96 - 1.67) | 0.95 (0.72 - 1.25) | 1.03 (0.78 - 1.37) | 0.64 |
| **HannumAge acceleration** |  |  |  |  |  |  |
| Vitamin A |  |  |  |  |  |  |
| Crude OR (95%CI) | 1.00 | 1.30 (1.02 - 1.67) ^*^ | 1.19 (0.93 - 1.53) | 0.96 (0.75 - 1.23) | 1.35 (1.05 - 1.72) ^*^ | 0.30 |
| Adjusted OR (95%CI) ^a^ | 1.00 | 1.34 (1.05 - 1.76) ^*^ | 1.31 (1.00 - 1.72) ^*^ | 1.06 (0.80 - 1.40) | 1.46 (1.10 - 1.93) ^**^ | 0.10 |
| Adjusted OR (95%CI) ^b^ | 1.00 | 1.37 (1.03 - 1.83) ^*^ | 1.30 (0.98 - 1.73) | 1.06 (0.79 - 1.43) | 1.36 (1.01 - 1.83) ^*^ | 0.28 |
| Vitamin B12 |  |  |  |  |  |  |
| Crude OR (95%CI) | 1.00 | 1.24 (0.97 - 1.59) | 0.94 (0.73 - 1.20) | 0.86 (0.68 - 1.11) | 0.86 (0.67 - 1.10) | 0.12 |
| Adjusted OR (95%CI) ^a^ | 1.00 | 1.21 (0.93 - 1.59) | 0.96 (0.74 - 1.26) | 0.85 (0.65 - 1.10) | 0.90 (0.69 - 1.10) | 0.06 |
| Adjusted OR (95%CI) ^b^ | 1.00 | 1.22 (0.92 - 1.63) | 0.97 (0.73 - 1.28) | 0.83 (0.63 - 1.10) | 0.89 (0.66 - 1.18) | 0.05 |

^a^ Adjusted for sex, PIR and race;

^b^ Additionally adjusted for smoking status, alcohol use, BMI and history of diabetes, cancer, stroke and CHD.

Abbreviations: OR, odds ratio; CI, confidence interval; PIR, poverty income ratio; BMI, body mass index; CHD, coronary heart disease.

**Supplementary Table 2** Association of quartiles of serum vitamins with epigenetic accelerated aging

|  | **Serum vitamins levels** | | | | ***P* for trend** |
| --- | --- | --- | --- | --- | --- |
|  | **Q1** | **Q2** | **Q3** | **Q4** |  |
| **PhenoAge Accel** |  |  |  |  |  |
| Vitamin A |  |  |  |  |  |
| Crude OR (95%CI) | 1.00 | 1.06 (0.85-1.32) | 0.86 (0.69-1.07) | 1.22 (0.98-1.52) | 0.289 |
| Adjusted OR (95%CI) ^a^ | 1.00 | 1.09(0.86-1.38) | 0.90(0.71-1.15) | 1.35(1.06-1.73) | 0.069 |
| Adjusted OR (95%CI) ^b^ | 1.00 | 1.10(0.85-1.41) | 0.89(0.69-1.15) | 1.29(1.00-1.68) | 0.02 |
| Vitamin B12 |  |  |  |  |  |
| Crude OR (95%CI) | 1.00 | 1.06(0.85-1.33) | 1.10(0.88-1.37) | 0.84(0.67-1.04) | 0.164 |
| Adjusted OR (95%CI) ^a^ | 1.00 | 1.06(0.84-1.34) | 1.12(0.88-1.41) | 0.81(0.64-1.03) | 0.152 |
| Adjusted OR (95%CI) ^b^ | 1.00 | 1.07(0.84-1.37) | 1.12(0.87-1.43) | 0.84(0.65-1.08) | 0.251 |
| **GrimAge Accel** |  |  |  |  |  |
| Vitamin A |  |  |  |  |  |
| Crude OR (95%CI) | 1.00 | 0.92(0.74-1.15) | 0.87(0.70-1.08) | 1.01(0.81-1.25) | 0.898 |
| Adjusted OR (95%CI) ^a^ | 1.00 | 0.88(0.68-1.12) | 0.87(0.68-1.12) | 0.96(0.75-1.24) | 0.774 |
| Adjusted OR (95%CI) ^b^ | 1.00 | 0.90(0.68-1.2) | 0.78(0.59-1.05) | 0.85(0.64-1.14) | 0.210 |
| Vitamin B12 |  |  |  |  |  |
| Crude OR (95%CI) | 1.00 | 0.78(0.63-0.98) * | 0.72(0.58-0.9) ** | 0.70(0.56-0.88) *** | 0.001 |
| Adjusted OR (95%CI) ^a^ | 1.00 | 0.84(0.66-1.07) | 0.74(0.58-0.95) * | 0.80(0.63-1.03) | 0.046 |
| Adjusted OR (95%CI) ^b^ | 1.00 | 0.78(0.59-1.03) | 0.70(0.53-0.93) * | 0.81(0.61-1.08) | 0.102 |

^a^ Adjusted for sex, PIR and race;

^b^ Additionally adjusted for smoking status, alcohol use, BMI and history of diabetes, cancer, stroke and CHD.

Abbreviations: OR, odds ratio; CI, confidence interval; PIR, poverty income ratio; BMI, body mass index; CHD, coronary heart disease.


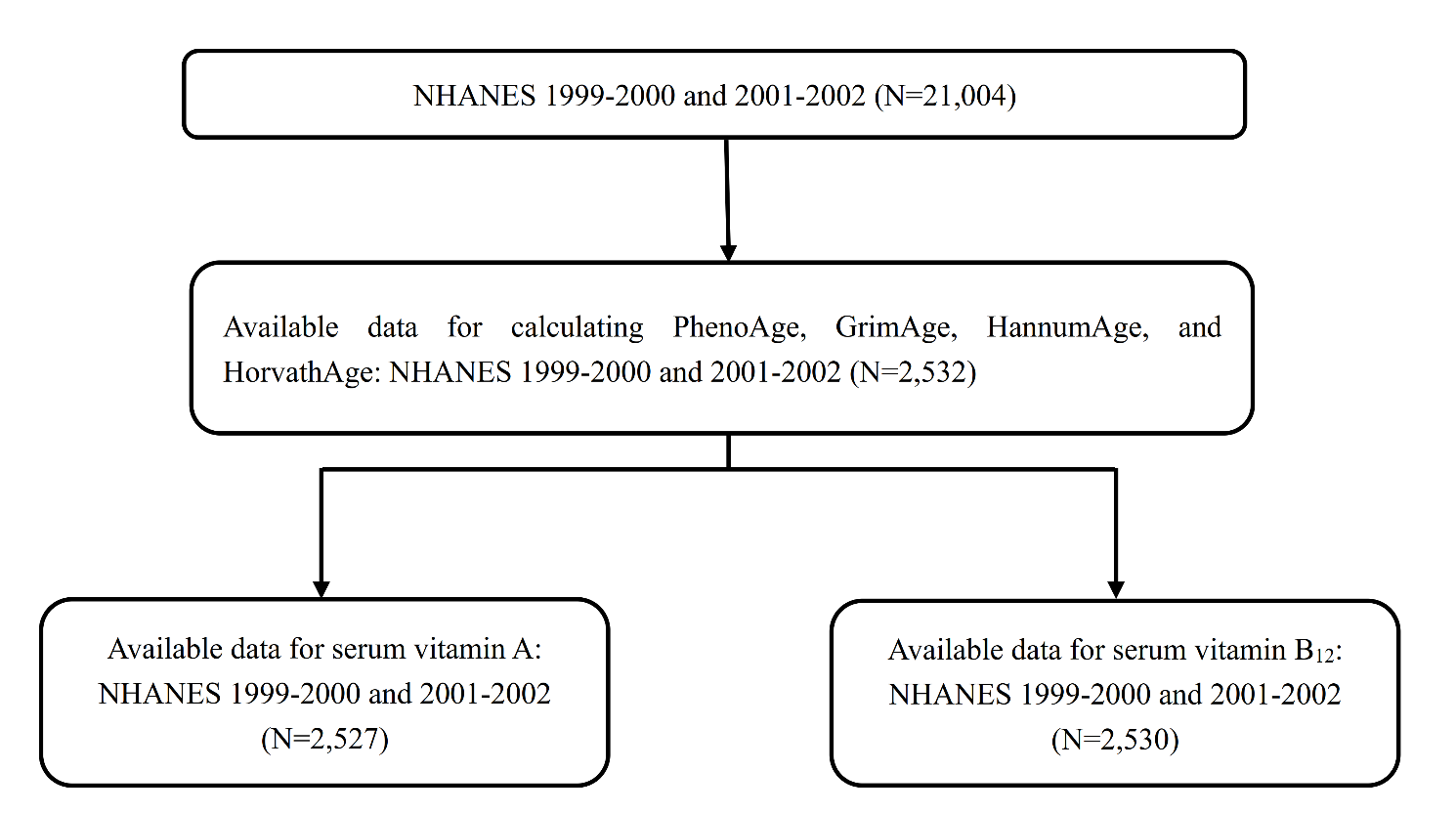


**Supplementary Figure 1** Flowchart showing selection of participants included in the main analysis from NHANES


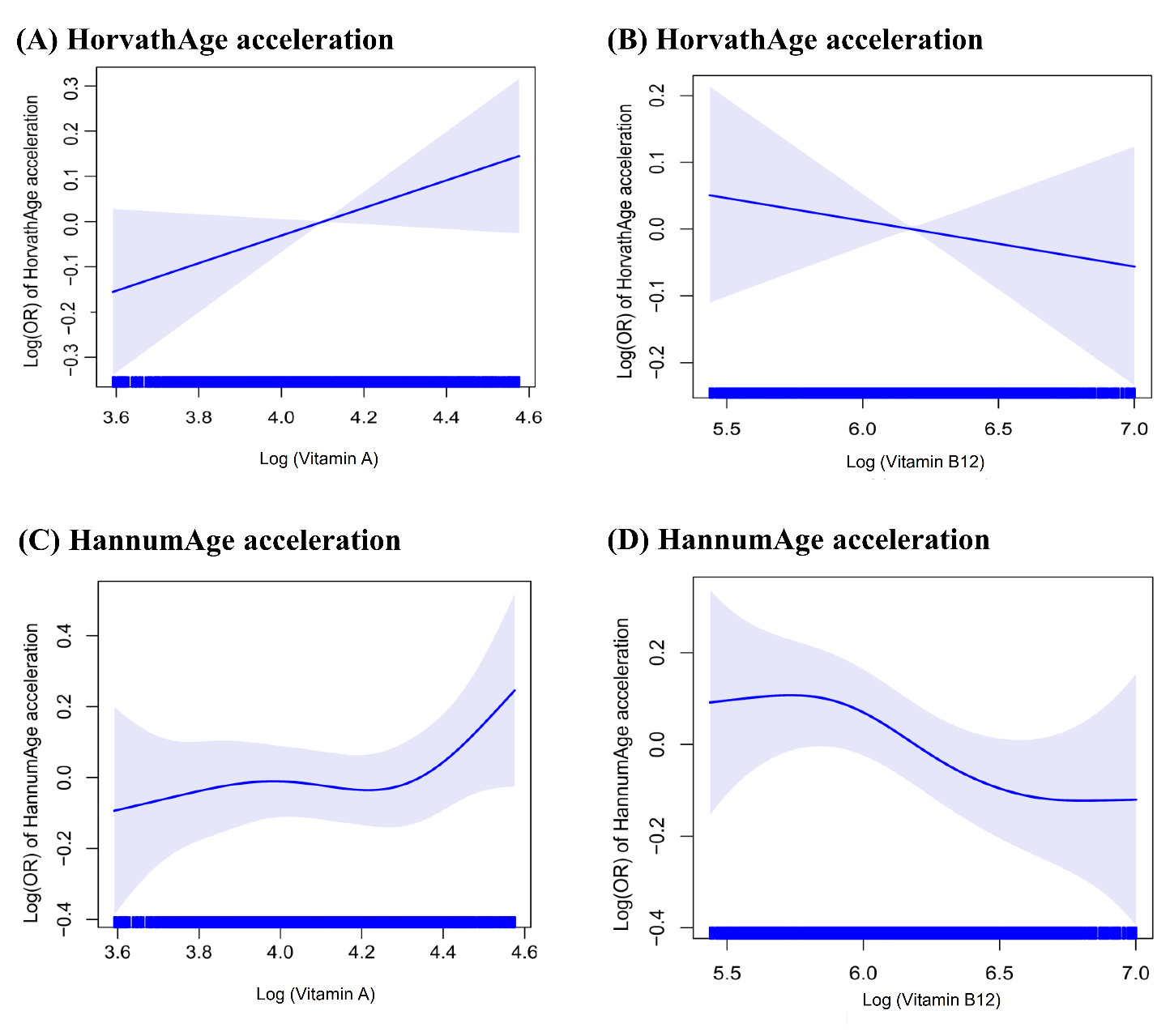


**Supplementary Figure 2** The nonlinear association of serum vitamin A and B12 concentrations with HorthAge acceleration and HannumAge acceleration
